# Adult teleost heart expresses two distinct troponin C paralogs: cardiac TnC and a novel and teleost-specific ssTnC in a chamber- and temperature-dependent manner

**DOI:** 10.1152/physiolgenomics.00074.2013

**Published:** 2013-07-23

**Authors:** Christine E. Genge, William S. Davidson, Glen F. Tibbits

**Affiliations:** ^1^Molecular Cardiac Physiology Group, Simon Fraser University, Burnaby, Canada;; ^2^Molecular Biology and Biochemistry, Simon Fraser University, Burnaby, Canada; and; ^3^Cardiovascular Sciences, Child and Family Research Institute, Vancouver, Canada

**Keywords:** myocardial contraction, evolution of troponin proteins, teleost atrial function

## Abstract

The teleost-specific whole genome duplication created multiple copies of genes allowing for subfunctionalization of isoforms. In this study, we show that the teleost cardiac Ca^2+^-binding troponin C (TnC) is the product of two distinct genes: cardiac TnC (cTnC, TnnC1a) and a fish-specific slow skeletal TnC (ssTnC, TnnC1b). The ssTnC gene is novel to teleosts as mammals have a single gene commonly referred as cTnC but which is also expressed in slow skeletal muscle. In teleosts, the data strongly indicate that these are two TnC genes are different paralogs. Because we determined that ssTnC exists across many teleosts but not in basal ray-finned fish (e.g., bichir), we propose that these paralogs are the result of an ancestral tandem gene duplication persisting only in teleosts. Quantification of mRNA levels was used to demonstrate distinct expression localization patterns of the paralogs within the chambers of the heart. In the adult zebrafish acclimated at 28°C, ssTnC mRNA levels are twofold greater than cTnC mRNA levels in the atrium, whereas cTnC mRNA was almost exclusively expressed in the ventricle. Meanwhile, rainbow trout acclimated at 5°C showed cTnC mRNA levels in both chambers significantly greater than ssTnC. Distinct responses to temperature acclimation were also quantified in both adult zebrafish and rainbow trout, with mRNA in both chambers shifting to express higher levels of cTnC in 18°C acclimated zebrafish and 5°C acclimated trout. Possible subfunctionalization of TnC isoforms may provide insight into how teleosts achieve physiological versatility in chamber-specific contractile properties.

teleost fish exhibit a wide variation in thermal tolerance and a capacity to acclimate to changing temperatures to cope with both longer term seasonal and acute temperature changes (43). The fish heart shows variable tolerance and plasticity to temperature (14), but there are many species-specific differences in these responses possibly correlating with habitat conditions and thermal preferences (39). The breadth of interspecies variation within teleost species is in stark contrast with cardiac muscle of endothermic mammalian species, making the temperature-induced adaptations of the contractile unit of teleost cardiac muscle intriguing.

The teleost heart is composed of four chambers arranged in series: venous sinus, atrium, ventricle, and bulbus arteriosus. The two contractile chambers acting as pumps are the atrium and ventricle, a simplified version of that seen in tetrapods. The atrium and the ventricle differ not only morphologically but also physiologically with distinct contractility characteristics. The end-diastolic volume in fish is largely determined by atrial contraction rather than central venous pressure as seen in mammals (9). This overall structure allows for increased reliance on changes in stroke volume to respond to greater hemodynamic loads. Because of this increased involvement of the atrium in driving ventricular filling and hence cardiac output, the fish-specific contractile properties of the atria and the ventricles are likely to be important in achieving a higher degree of physiological versatility (18).

Temperature is a striking environmental condition that greatly impacts cardiac contractility in ectothermic species. Factors such as heart rate, cardiac output have all been shown to fluctuate in species such as salmonids with changing temperature. With decreased temperature, the Ca^2+^ sensitivity of the cardiac contractile unit decreases in both mammals (25) and fish (8) despite myofilament Ca^2+^ sensitivity being a critical factor in regulating cardiac contractility. It has been postulated that ectotherms require heightened myofilament Ca^2+^ sensitivity to maintain cardiac function through changing environmental conditions. Skinned salmonid cardiomyocytes have been shown to have a significantly higher Ca^2+^ sensitivity at any given temperature (over the measured range of 2–28°C) than those from mammals (8). A key regulator of myofilament Ca^2+^ sensitivity, the cardiac troponin (cTn) complex, is made up of three proteins (cTnC, cTnI, cTnT). TnC, the Ca^2+^ binding protein that initiates myocyte contraction, has been shown to play a role in enhancing myofilament Ca^2+^ sensitivity in fish (20). TnC consists of two globular domains connected by an α-helical linker with each domain having two EF-hand Ca^2+^ binding sites. In mammalian fast skeletal TnC (fsTnC), all four EF hand sites bind Ca^2+^ with affinities that are physiologically relevant. The COOH-terminal sites are considered high-affinity Ca^2+^/Mg^2+^ sites and are known to be structurally integral to anchoring TnC to the troponin complex, while the NH_2_-terminal domain sites are relatively low affinity but Ca^2+^ specific. cTnC possesses only one low-affinity Ca^2+^ binding site (site II) as site I has been rendered nonfunctional by amino acid substitutions that preclude Ca^2+^ coordination. In cardiac muscle Ca^2+^ binding to site II initiates a complex change in the interrelations of the troponin molecules on the thin filament initiating force production of the contractile element, making cTnC a key regulator of myofilament Ca^2+^ sensitivity (37).

The importance of the role of cTnC is demonstrated by its high degree of sequence conservation across phylogenetically diverse groups of organisms and millions of years of evolution. In fact, across mammals, cTnC amino acid sequences are almost completely identical while mammalian sequences relative to that of trout show 92% amino acid identity (22). Because of the key role of this protein in cardiac physiology, modifications in the sequence of amino acids resulting in changes in function of TnC can alter the entire contractile reaction of the myocyte. Despite the highly conserved nature of TnC, subtle variations are seen as interspecies orthologs and intraspecies paralogs between tissues. In trout cTnC, specific amino acids have been shown to be responsible for the increased Ca^2+^ affinity seen relative to mammals, including Asn2, Ile28, Gln29, and Asp30 (NIQD). These residues are hypothesized to allosterically affect the ability of site II to bind Ca^2+^ (24). This increased Ca^2+^ sensitivity is observed despite the fact that fish cTnC are temperature sensitive. These amino acid substitutions enable greater Ca^2+^ binding at lower temperatures than would otherwise be possible (23).

However, with the teleost-specific whole genome duplication (31), the possibility exists that multiple isoforms of genes exist that may be expressed differentially on a temporal or spatial scale or in response to environmental factors such as temperature. While most genes are lost after a duplication event, tissue specific expression may lead to increased retention of multiple isoforms since paralogs can divide up ancestral function (subfunctionalization) or assume new functions (neofunctionalization) (15). In zebrafish embryos two separate paralogs have been identified as being comparable to mammalian cTnC: one primarily expressed in the heart (TnnC1a or cTnC) and the other in slow skeletal muscle (TnnC1b or ssTnC) (41). One important note on nomenclature is that TnnC1a and TnnC1b are consensus terms used by the Zebrafish Community's information network (http://www.zfin.org), while the terms cTnC and ssTnC are consensus terms in the mammalian literature and both are used in this paper. These genes are both located on chromosome 23 in zebrafish (cTnC, 4055544–4060124; ssTnC, 19608998–19611413) but display independent gene expression patterns both temporally and spatially between tissues. This expression localization pattern suggests differences in functionality between the two paralogs. For this reason, these genes are normally referred to by the location in which they are the most expressed, ssTnC and cTnC. However, thus far this pattern has only been seen in embryo with no information on adult zebrafish paralog distribution, or in fact the presence of any orthologs in any other teleost species.

Through phylogenetic analysis of existing fish genome sequences, we demonstrate that ssTnC is not only present in zebrafish (as seen in embryos) but broadly across teleosts species (12, 16, 48). Using adult zebrafish and rainbow trout as models, we examined paralog gene expression tissue localization patterns as well as temperature acclimation effects to begin to examine the relevance of three fish-specific paralogs of TnC. Our focus was on the two paralogs expressed in cardiac tissue and their relation to atrio-ventricular differences in contractility (18). We show that the ssTnC and cTnC transcripts are expressed in a chamber-specific manner in both the zebrafish and rainbow trout hearts, indicating a possible involvement in functional differences in the atrium and ventricle like other chamber specific isoforms of contractile proteins [e.g., myosin heavy chain and myosin light chain (30, 46)]. Moreover, we found both chambers have a unique response of relative paralog expression to an environmental perturbation: temperature acclimation.

## METHODS

### 

#### Sequence and phylogenetic analyses.

All available TnC gene sequences homologous to zebrafish cTnC or ssTnC were found using the National Center for Biotechnology Information (Bethesda, MD) nonredundant protein database (http://www.ncbi.nlm.nih.gov/guide/proteins/), the Ensembl Genome Browser (Wellcome Trust Genome Campus, Hinxton, Cambridge, UK) (http://uswest.ensembl.org/index.html), and GenBank (http://www.ncbi.nlm.nih.gov/genbank/). BLAST searches (2) were set up for several species expressed sequence tag (EST) databases against zebrafish cTnC (TnnC1a) and ssTnC (TnnC1b) sequences (GI:28822162 and GI:50344823, respectively).

Multiple amino acid sequence alignments were performed using Multiple Sequence Comparison by Log-Expectation (13) via MEGA5 (Molecular Evolutionary Genetics Analysis 5.0, Ref. 44). The evolutionary histories for these amino acid sequences were inferred using the maximum likelihood algorithm (MEGA5). Analyses were based on the JTT-matrix model (29), the optimal model of evolution built on the Akaike Information Criterion (27). For maximum likelihood, the bootstrap consensus trees were inferred from 500 replicates. There were a total of 161 residues in the final dataset.

#### Animals and cold acclimation.

All animal experiments were approved by the Simon Fraser University Animal Care Committee (protocol number 989K-90). Adult zebrafish purchased from a local supplier were maintained at a 12 h/12 h light-dark photoperiod at 28°C in dechlorinated water in a flow-through tank and fed ad libitum (Nutrafin Max; Hagen, Baie d'Urfé, QC, Canada). Rainbow trout were held in an outdoor facility in dechlorinated water and fed ad libitum. For the thermal acclimation group of adult zebrafish, after being held at 28°C for 2 wk in flow-through tanks, the temperature was decreased by 2.5°C/wk until the end-point temperature of 18°C was reached. Fish were then held at this temperature for 3 wk. Trout were allowed to seasonally shift with ambient temperature and light cycles, but cardiac and skeletal muscle data were collected at a temperature of 5°C during the winter months and 15°C during the summer.

Zebrafish were killed via cold shock followed by decapitation. Trout were killed by a swift blow to the skull followed by severing the spinal cord. Cardiac tissue from whole heart and skeletal tissue from the lateral line was taken from a first group of fish (28°C in zebrafish and 5°C in trout). For the remaining warm- and cold-acclimated fish, the heart was removed via a small triangular section that was excised from the zebrafish along the gill arch to the head. From this section we teased out the whole heart by grabbing onto the bulbus arteriosus under a dissecting scope. After the bulbus arteriosus was removed, the atrium and the ventricle were physically separated with scissors (40). In the trout, the larger size allowed for more traditional dissection through the ventral side. All samples were placed immediately into cold Trizol (Invitrogen, Burlington, ON, Canada). Seven fish were pooled for each individual sample and then each sample was frozen at −80°C and stored at this temperature until use.

#### RNA analysis.

Total RNA was extracted from each sample using Trizol followed by RNeasy kit (Qiagen, Mississauga, ON, Canada) according to the manufacturer's instructions. Samples were quantified spectrophotometrically at 260 nm before storage at −80°C. We assessed RNA purity by measuring absorbance at 280 nm to assess protein contamination, with any samples having the 260/280 ratio falling outside the range of 1.9–2.1 being a criterion for being discarded. Genomic DNA removal and reverse transcription of RNA samples were done using the Qiagen Quantitect Reverse Transcription kit according to the manufacturer's instructions with each reaction scaled to 0.5 μg RNA.

Real-time quantitative PCR (qRT-PCR) analysis was performed on a Bio-Rad CFX96 Touch Real-Time PCR System (Bio-Rad, Mississauga, ON, Canada) using the following conditions: an initial denaturation for 10 min at 95°C followed by 40 cycles of 15 s denaturation at 95°C, 30 s annealing at optimal primer temperature (Table 1), and 36 s extension at 72°C. Samples were assayed in duplicate in a 20 μl reaction volume containing 10 ng cDNA, 12 μl SsoAdvanced SYBR Green Supermix (Bio-Rad) and 0.25 μmol of each primer. Negative controls (no template or selected untranscribed RNA) were run as well to ensure the absence of contamination. Select control samples were reassayed to ensure no significant difference between assays.

**Table 1.** Primers used for quantitative RT-PCR analyses

Gene	Accession Number	Primer F	Primer R	Product Size, bp	Tm, °C
ZF cTnC	AF434188	AAGCAGCGGCAGAGCAAC	GCTCTTCAGGGGTAGGGTTC	153	59
ZF ssTnC	NM_001002085	AGCAGCGGTGGAGAACTTG	GCTCTTCTTGAGTGGGGTTTT	152	59
ZF β-actin	AF057040	TTCTGGTCGGTACTACTGGTATTGTG	ATCTTCATCAGGTAGTCTGTCAGGT	170	59
ZF ef1α	AY422992	TGGAAATTCGAGACCAGCA	GGTCTGTCCGTTCTTGGAGA	168	59
RT cTnC	AY281129	CTACAAAGCAGCGGTAGAGC	CAGTTCCACTGCCATCTTCA	203	59
RT ssTnC	BX863663.3	AAGCAGCGGTTGAGAACTTA	GGAACTCATCAAAGTCTACTGTCC	216	59
RT β-actin	AF157514	TTCAACACCCCTGCCATGTA	CGTCAGGGTCTTCATCAGGT	204	59
RT ef1α	NM_001124339	CCCCTCCAGGATGTCTACAAA	CACACGGCCCACGGGTAC	210	59

F, forward; R, reverse; Tm, melting temperature; ZF, zebrafish; cTnC, cardiac troponin C; ssTnC, slow skeletal TnC; ef1α, elongation factor 1 alpha; RT, rainbow trout.

Analysis was performed according to the ΔΔCt method using the geometric mean of two distinct housekeeping genes to normalize the data (45). β-Actin was originally chosen as an appropriate reference gene for zebrafish (6) and confirmed by both dissociation curves and the stability of Ct values across conditions and samples. The second gene, elongation factor-1 alpha (ef1α), was measured using two primer sets, one spanning exons 3 and 4, and the second spanning exons 5 and 6, allowing for the comparison of values from each primer set to check for RNA stability (Ref. 28, second gene set data not shown). Specific primers for each gene were designed using a combination of Primer3 (38) and Oligonucleotide Properties Calculator (32) to amplify a single product (using zebrafish published sequences, see Table 1), as checked by regular PCR and dissociation curve analysis post-real-time PCR. Products were confirmed via sequencing (CMMT/CFRI DNA Sequencing Core Facility, Vancouver, BC, Canada). All data are presented as mean values ± SE and are corrected for by using the geometric mean of β-actin and ef1α 1, then expressed relative to the isoform with either lower expression (Fig. 4) or ssTnC (Fig. 5). Significance was determined between chambers by *P* values < 0.05 by unpaired Student's *t*-tests.

## RESULTS

### 

#### TnC sequence variation.

**Fig. 1.**
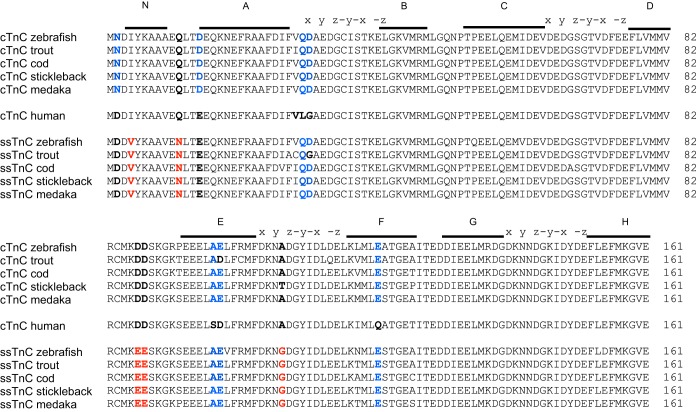
Sequence comparison of representative slow skeletal troponin C (ssTnC) and cardiac troponin C (cTnC) sequences from fish and human. The Ca^2+^-coordinating positions in each EF-hand site are shown above the sequences (x y z-y-x -z), and the helices are labeled (N, A, B, C, D, E, F, G, H). Blue residues are fish-specific; red are fish ssTnC specific.

High sequence similarity shows TnC is very well conserved across vertebrates. However, between fish and mammalian sequences there are several variations. While there are 16 amino acid sequence differences (out of a total of 161 residues) between zebrafish and mammalian cTnC, there are 19 sequence differences between zebrafish ssTnC and mammalian cTnC (Fig. 1). There are 18 amino acid sequence substitutions between the zebrafish paralogs ssTnC and cTnC. There is >93% identity (at the amino acid level) between cTnC orthologs as well as between ssTnC orthologs, while there is between 87–89% identity between cTnC and ssTnC paralogs across teleosts (select species shown in Fig. 1). Lamprey displays much lower sequence identity than that seen between teleosts (alignment not shown between teleosts and lamprey). The majority of TnC residues critical for Ca^2+^ binding (17, 19) are conserved across vertebrates and likewise between cTnC and ssTnC. Like cTnC, ssTnC appears to have only site II as a functional Ca^2+^ binding domain in the NH_2_ terminus based on the coordinating residues (19). Meanwhile, sites III and IV in the COOH-terminal domain are conserved across all species in both ssTnC and cTnC. These sites in all species examined have the X Y Z -Y -X Z sites with residues possessing either a carboxyl or hydroxyl group with the ability to contribute an oxygen atom to coordinate Ca^2+^ (19, 21).

In the NH_2_-terminal domain, site II is a region of high sequence conservation due to its importance for the functioning of TnC (22). Site I of all cTnC orthologs displays lower sequence similarity than seen in fsTnC. The insertion of a valine at residue 28 and the replacement of chelating residues Asp29 and Asp31 in all vertebrate cTnCs relative to fsTnC are the reasons for the inability of site I to bind Ca^2+^ (19). This is seen in site I of both teleost ssTnC and cTnC. In both paralogs, certain residues found in teleost hearts such as Gln29 and Asp 30 appear to be teleost-specific traits, but residue 28 shows some variability with Val as seen in mammals and Ile as seen in trout (21). Other residues, such as Asp2 and Glu14, are found in all mammalian cTnC and fish ssTnC but not fish cTnC. Still others, such as Val4, Asn11, Glu87, and Glu88 appear to be present only in teleost ssTnC.

#### Phylogenetic analysis.

**Fig. 2.**
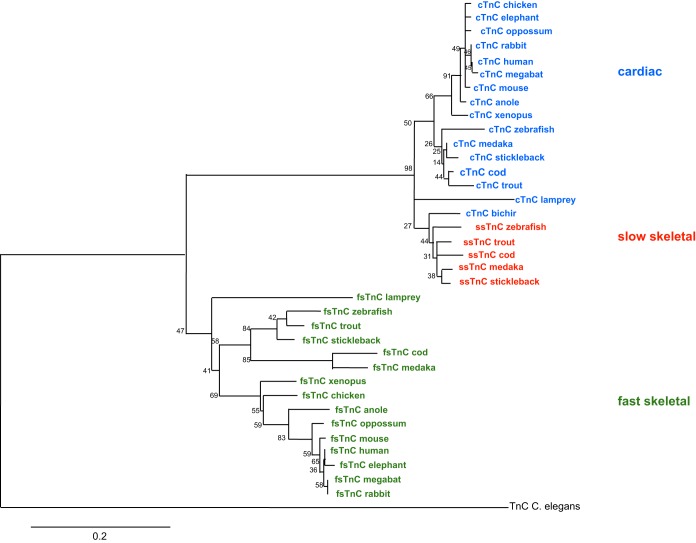
Phylogenetic tree generated by the maximum likelihood comparison of TnC genes sequences across vertebrates demonstrating a 3rd fish-specific paralog. The evolutionary history was inferred using the maximum likelihood method based on the JTT-matrix model (29). The bootstrap consensus tree was inferred from 500 replicates with the percentage of replicate trees in which the associated taxa clustered together is shown next to the branches. The tree is drawn to scale, with branch lengths measure in the number of substitutions per site. The tree is rooted with *Caenorhabditis elegans* (invertebrate) TnC. The analysis involved 37 amino acid sequences of representative vertebrate TnC sequences compiled from NCBI and Ensembl. Evolutionary analyses were conducted in MEGA5 (44).

**Fig. 3.**
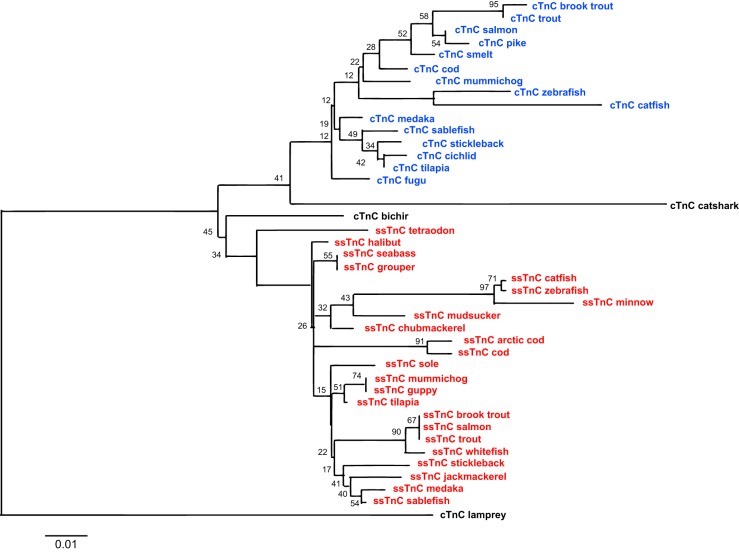
Evolutionary history of cTnC and ssTnC amino acid sequences across fish. The evolutionary history was inferred using maximum likelihood method, based on the JTT-matrix model (29). The bootstrap consensus tree was inferred from 500 replicates with the percentage of replicate trees in which the associated taxa clustered together is shown next to the branches. Values represent bootstraps expressed as percentage. Trees are drawn to scale, with branch lengths indicating the number of substitutions per site and rooted with lamprey cTnC. Evolutionary analyses were conducted in MEGA5 (44).

Due to the length of time since the divergence among vertebrate lineages, the phylogenetic analyses were made based on amino acid sequences rather than nucleotide sequences, in which multiple changes at the third codon positions are likely to have occurred. Tree topologies were consistent with accepted relationships among vertebrates (22) both in maximum likelihood analysis (Figs. 2 and 3) and Bayesian analysis (tree not shown). The values reflect the high degree of conservation of TnC with only 18 of 161 residues being variable between isoforms. However, as shown in Fig. 2, two distinct TnC clades are seen across vertebrates, with greater variation in the fish lineages in both fsTnC and cTnC. As well as the two traditional groupings across vertebrates, fish cTnC is split into two branches labeled as ssTnC and cTnC. For several species of teleost, such as several representative salmonids and the grouper, we have reannotated them as ssTnC as opposed to cTnC as listed in NCBI based upon their grouping and sequence similarity (Fig. 3). A more in-depth examination of teleost (jawed, bony fish) TnC sequences shows that both paralogs have diverged from the TnC in the jawless lamprey, which is ancestral to teleosts and tetrapods. The basal positions of both the condrichthyes (e.g., catshark) and the ray-finned fish (e.g., bichir) TnC sequence are also consistent with the ancestral state.

#### Tissue-specific expression patterns.

**Fig. 4.**
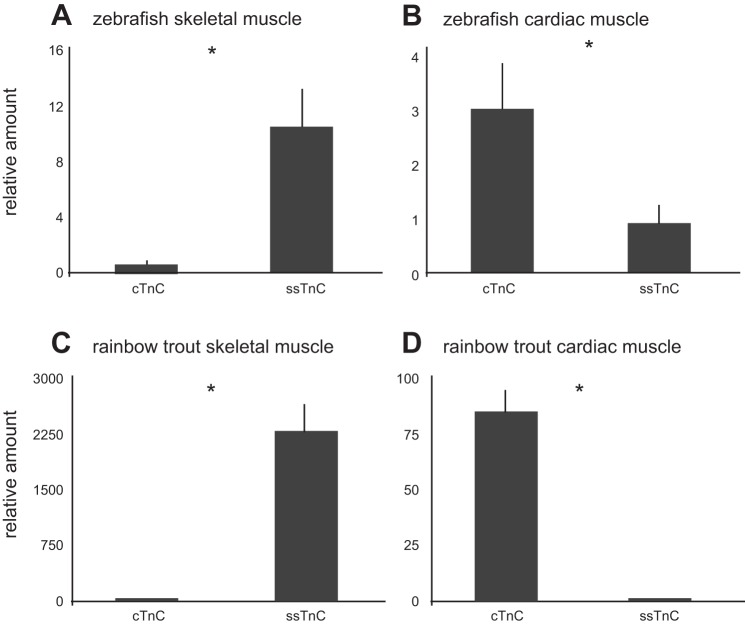
Quantitative real-time PCR was used to determine tissue specific differences in relative mRNA levels of TnC paralogs in adult zebrafish at 28°C and adult rainbow trout at 5°C. Values are expressed as mean (*n* = 8) of mRNA levels normalized to geometric mean of β-actin and ef1α by the ΔΔCt method (51) and are then expressed as fold-difference from the lower expression isoform. Vertical error bars represent SE. Note the scaling of the *y*-axis in skeletal muscle (*A*) is 4-fold larger than cardiac muscle in zebrafish (*B*), and skeletal muscle (*C*) is 30-fold larger than cardiac muscle (*D*) in trout. *P < 0.05 (Student's *t*-test).

Quantitative real-time PCR (qRT-PCR) was used to quantify the tissue-specific gene expression of ssTnC and cTnC in adult zebrafish at 28°C and adult rainbow trout at 5°C. The dominant transcript measured in zebrafish skeletal mixed muscle with qRT-PCR was ssTnC with minimal amounts of cTnC (Fig. 4*A*). However, in zebrafish cardiac tissue while cTnC was the dominant paralog (*P* = 0.009), both genes were highly expressed with the expression of cTnC only threefold greater than that of ssTnC (*P* = 0.02) (Fig. 4*B*). In rainbow trout cardiac muscle cTnC was the dominant paralog (Fig. 4*C*), while in skeletal muscle ssTnC was almost exclusively expressed (3,000-fold greater than cTnC) (Fig. 4*D*).

**Fig. 5.**
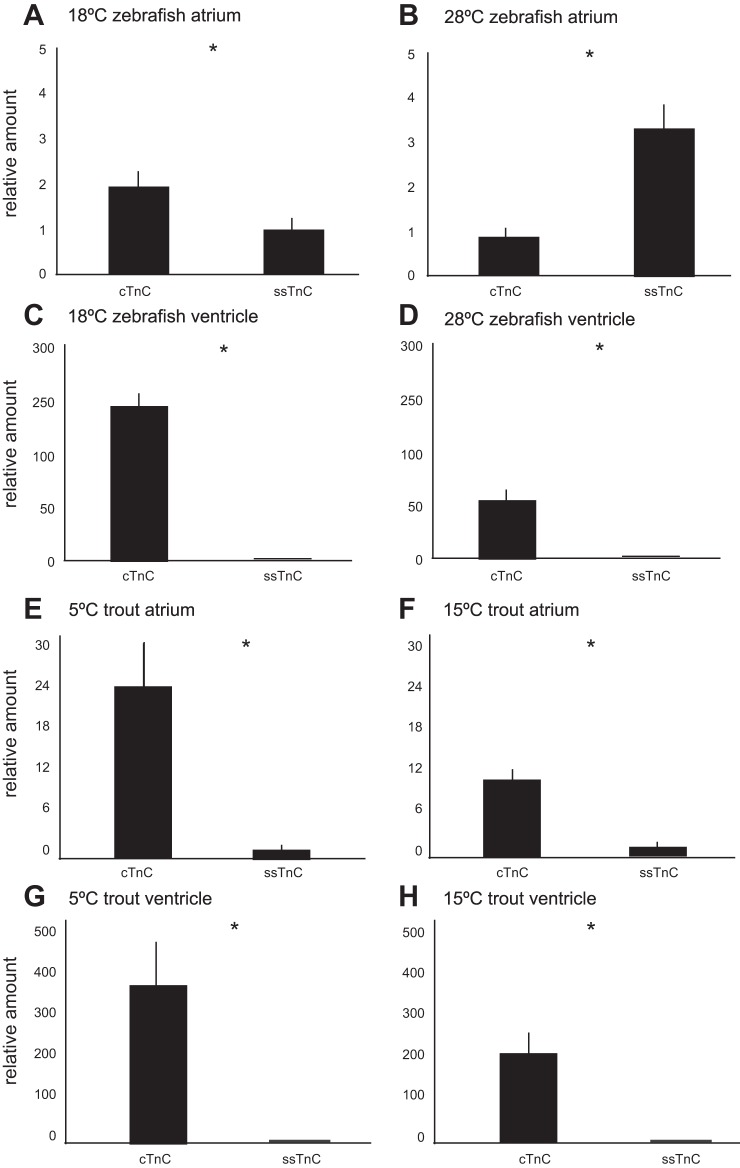
Quantitative real-time PCR was used to determine tissue-specific differences in relative mRNA levels of TnC paralogs in adult zebrafish and trout. *A*: atrial tissue at 18°C, *B*: atrial tissue at 28°C, *C*: ventricular tissue at 18°C, *D*: ventricular tissue at 28°C in adult zebrafish; and in adult trout *E*: atrial tissue at 5°C, *F*: atrial tissue at 15°C, *G*: ventricular tissue at 5°C, *H*: ventricular tissue at 15°C. Values are expressed as mean (*n* = 8) of mRNA levels normalized to the geometric mean of β-actin and ef1α by ΔΔCt method (51) and then relative as fold difference to ssTnC which was set to 1. Vertical error bars represent SE. *P < 0.05 (Student's *t*-test).

To clarify further the division of expression in cardiac muscle, gene expression was quantified in the two main chambers of the heart, the atrium and ventricle (Fig. 5). We determined that in adult zebrafish the cTnC transcript was expressed 150-fold more than ssTnC in the ventricle (*P* = 0.001) when both were normalized to β-actin. Conversely, in the atrium, ssTnC transcript levels were significantly higher (2-fold) than cTnC (*P* = 0.004).

In 5°C trout, the cTnC transcript was the dominant paralog in both the atrium and ventricle, though the atrium had only a 23-fold difference in cTnC relative to ssTnC, while a 360-fold difference in cTnC relative to ssTnC was observed in the ventricle (Fig. 4*E*). In both species β-actin and ef1a transcript levels were comparable in both chambers.

#### Cold acclimation gene expression patterns.

Zebrafish acclimated at colder temperatures (18°C) displayed significantly increased relative expression of cTnC in both chambers of the heart (Fig. 5). In the ventricle, cTnC went from 150-fold higher than ssTnC at 28°C to 300-fold higher in 18°C (*P* = 0.0004). In the atrium, cTnC went from twofold lower than ssTnC at 28°C to 1.5-fold higher (*P* = 0.005) at 18°C. Again, β-actin and ef1a transcript levels did not significantly change with temperature in either chamber.

The profile of both paralogs in either chamber still differed significantly from the 5°C rainbow trout. To clarify whether this difference was simply due to decreased temperature or a phylogenetic difference, rainbow trout were seasonally acclimated to 15°C. While cTnC was still the dominant isoform in both chambers, ssTnC expression did increase with higher temperature.

## DISCUSSION

The critical role of troponin C in the regulation of contraction has resulted in this protein being highly conserved across a wide range of species. Previously, the fast skeletal and cardiac TnC genes were thought to be the only paralogs resulting from a genome duplication event prior to the vertebrate radiation (22, 36). However, beyond the typical groupings of cardiac and fast skeletal TnC, zebrafish have two apparent paralogs of what has been annotated as cTnC (41). In the zebrafish embryo, these two paralogs exhibited a tissue specific pattern of distribution in cardiac and slow skeletal muscle, though functionally either would allow for proper contraction of the heart in embryo (41). These paralogs may have arisen by a tandem gene duplication, in which case they should be on the same chromosome. Alternatively, the paralogs may be the result of the teleost-specific whole genome duplication (34), in which case they should be observed on the homologous chromosomes. Our search of genomic databases revealed clearly that both cTnC (TnnC1a) and ssTnC (TnnC1b) occur in virtually every teleost genome examined to date. Moreover, both paralogs appear on the same chromosome in the limited species where genomic location is available for (zebrafish, chromosome 23; stickleback, group XII; medaka, chromosome 7). This suggests that the paralogs arose from a tandem gene duplication that occurred in the common ancestor of the teleosts.

### 

#### Phylogenetic analysis of TnC.

More extensive genome sequence information has become available since previous work on the evolution of TnC (22). These additional species genomic builds allowed us to identify potential TnC orthologs based on sequence similarity to zebrafish cTnC and ssTnC from ESTs. Signature residues of the ssTnC clade, which distinguish these sequences from the cTnC clade (specifically Asp2, Val4, Asn11, Glu14, Glu87/88, Gly108; Fig. 1), appear in all teleosts. We posit that the conservation of all these unique residues in ssTnC representatives indicates that these genes are two different paralogs. For all teleosts in which two cTnC sequences were found, representing species that exist over a broad range of different environmental conditions (cold vs. warm, sedentary vs. active), these residues that we believe to be unique to ssTnC were clearly identified. While no variation exists in the sequence of the regulatory Ca^2+^ binding site (*site 2*) including the six coordinating residues, variation does exist in *residue 2*, one of four residues implicated in the functional difference between mammalian and fish cTnC Ca^2+^ binding affinity (21, 24). With ssTnC resembling mammalian cTnC (possessing the polar acidic aspartic acid) rather than fish cTnC (polar uncharged asparagine) in *residue 2*, a possible difference in Ca^2+^ affinity may exist. Functional characterization of binding affinities of both isoforms in zebrafish will be necessary for the physiological impact of these residue variations to be determined.

Phylogenetic analysis of representative vertebrate species shows the typical division of two clades (fsTnC and cTnC) with the addition of a third fish-specific clade (ssTnC, Fig. 2). The maximum likelihood phylogeny still reflects the accepted phylogeny of fish species and thus does not reveal anything novel about evolutionary adaptations within fish in TnC gene structure. While cTnC forms a separate clade from fsTnC supported by higher bootstrap value (98% support for the cTnC-specific clade), the values shown for the separate cTnC paralogs have relatively low support. Within the clades all bootstrap values demonstrate low support, even between phylogenetically diverse species such as fish and mammals. In this study the cTnC sequence, as in previous studies, is fundamentally similar across species of teleosts. With fewer species sequences available in previous work, this cTnC sequence similarity has led to the assumption that the NH_2_-terminal sequence is completely conserved (22, 47). Hence low support from bootstrapping is reflective of the high sequence similarity of TnC isoforms and is not likely to be indicative of an inaccurate relationship. Moreover, Bayesian analysis (tree not shown) supported the overall configuration of two independent isoforms in teleosts only (cTnC and ssTnC) with slightly higher support from posterior probabilities taking into account prior probabilities as well as likelihood of placement. As mentioned above, the conservation of ssTnC-specific residues relative to those in cTnC across teleosts supports the hypothesis of these two genes being separate paralogs. Several species included in previous fish TnC work had to be excluded from our analyses because several distinguishing residues could not be sequenced in the extreme NH_2_ terminus of these ssTnC/cTnC genes (22).

The evolutionary timing of the appearance of two isoforms of cTnC may be inferred from the phylogenetic relationships. While genetic variability may be generated via point mutations, it is probably large-scale genomic duplication events that are primarily responsible for the diversity seen across vertebrate gene families (35). Initially a duplication event provides organisms with two sets of genes presumably carrying redundant functions. Redundant genes are normally lost or reduced to pseudogenes, but selective pressures may conserve one of the copies, while the other may be relieved from these constraints and rapidly evolve. This could result in each copy assuming only a portion of the functions of the ancestral gene (subfunctionalization) or one copy developing an entirely new function (neofunctionalization) (15). The duplicated TnC genes are thought to have subfunctionalized into fsTnC and cTnC sometime following the split between urochordates, where a primitive heart appeared, and agnathans with the basic chamber structure of a fish heart (13), where distinct fsTnC and cTnC appear (22).The cTnC from lamprey, a representative agnathian, is clearly divergent from all other fish species. A single copy of cTnC appearing in both lamprey and across tetrapods appears to be consistent with the ancestral state.

Basal ray-finned fish such as the bichir can help with the understanding if these two genes are products of the teleost-specific genome event. Polypteriformes diverged from teleosts prior to the 3R whole gene duplication event specific to teleosts (7), which occurred ∼440 million years ago (3, 34). While bichir TnC appears to be grouped in with the ssTnC based on the sole sequence available, there is still only one copy of a cTnC-like gene. This bichir cTnC sequence has some residues matching teleost cTnC (e.g., Asp2), some matching teleost ssTnC (e.g., Glu87/88), and others matching teleost ssTnC or tetrapod cTnC (e.g., Val9). This mix of teleost and tetrapod features seen in the bichir sequence is not exclusive to TnC but has been seen in other proteins involved with E-C coupling [e.g., RyR (10)]. Bichir thus provides an interesting case of the preteleost duplication ancestral state, with the current two cTnC paralogs possibly representing a mixture of the ancestral characteristics. This suggests multiple cTnC paralogs are teleost specific, but lacking the availability of characterization of the full genome of a species in the Polypteriformes, such as the bichir or the gar [current genome projects are ongoing such as EnsemblPre! for the spotted gar (*Lepisosteus oculatus*) or Vertebrate TimeCapsule for the gray bichir], this may be a premature statement.

While the maximum likelihood analysis shows both bichir and catshark in basal positions to teleost TnC paralogs, their relationship to each other is not completely clear. Bayesian inference shows bichir in particular to be much more strongly grouped with ssTnC than maximum likelihood does (data not shown). Bayesian posterior probabilities and bootstrap support values often have observed discrepancies (26) with posterior probabilities tending to be more generous than bootstraps, similar to what is seen in the TnC analysis. Bootstrap support merely helps predict whether the same result would be seen if more data were collected, not whether the result is correct. Posterior probabilities refer to the probability that the result is correct given the hypothesis or prior information given. More data (further sequences) will decrease the impact of priors and make Bayesian analysis stronger and allow for possible resolution of basal species. However, until these complete genomic and chromosome location data are determined, the available information is suggestive of these paralogs being the result of an ancestral tandem gene duplication persisting only in teleosts.

Even in a protein as highly conserved as TnC, the presence of three genes in teleosts provides evidence of multiple paralogs in the fish lineage. This demonstrates the complications that the additional gene and genome duplications may bring, especially for research relying on cross-species comparisons for traditional techniques of determining the amount of protein translated such as Western blotting. Nonspecies-specific antibodies are unlikely to account for all isoforms and lead to inaccurate quantification of protein expression. Furthermore, generating antibodies to discern the difference between cTnC and ssTnC would be difficult due to the high degree of sequence similarity and the low feasibility of finding unique epitopes. In fact, with trout seven TnI isoforms were all detected by one antibody, and even semiquantitative values could not be inferred (1). The presence of multiple isoforms underlies just one of the issues in accurately quantifying proteins in comparative models and, hence, made it an unattractive option in our current study. These differences in localization are then solely based on mRNA data due to lack of confidence in accurate quantification of protein translation. This genomic information may provide information about the theoretical status of cellular proteins, but proteomic information describes the actual content, which ultimately determines the phenotype. Changes in the transcription of genes are still key to modifying the proteome, and the patterns identified here in zebrafish should be considered representative.

#### Paralog localization.

We found differential expression in the transcript levels of ssTnC and cTnC in the adult zebrafish heart (Fig. 4). The three different isoforms of TnC (fsTnC, cTnC, ssTnC) found in zebrafish are labeled based on their localization in embryo (41), which in turn reflects the classification of different types of muscle. However, in the adult zebrafish gene expression data provided in this study, cardiac muscle does not express one isoform, but rather two. The ratio of ssTnC/cTnC in zebrafish whole heart is suggestive of atrium to ventricle mass ratio [typically between 0.2 and 0.3 (33)]. Our data explore this further, showing ssTnC is actually predominant in the atrium, whereas cTnC is predominant in the ventricle, but only in zebrafish at warmer temperatures. Even in trout atrium, ssTnC transcript levels are much more pronounced than in ventricle, especially at warmer temperatures. This differential gene expression in these two chambers may be reflective of the unique contraction of the fish atrium and ventricle relative to mammals (18). In mammals, gene expression of sarcomeric proteins is guided both temporally and spatially to contribute to the overall chamber-specific phenotype. Different subsets (paralogs) of sarcomeric protein expression such as myosin heavy chain and myosin light chain confer differing contractile properties to the atrium and the ventricle.

#### Phylogenetic different paralog localization.

While the chamber-specific profile in zebrafish suggests chamber-specific contractility differences may be guiding expression patterns of TnC paralogs, we did not see a similar pattern in the cold-water fish measured: rainbow trout in which both chambers predominantly express cTnC. Although both these fish are teleosts, they have distinct phylogenetic histories with different environmental constraints. The salmonid trout are much larger and more active and live at far colder temperatures than the cyprinid zebrafish. Species have specific expression patterns of certain genes to allow them to be innately suited to their environmental conditions, which may not be readily predictable by only looking at a single variable [i.e., species-specific thermal compensation in mitochondrial gene expression (5), hypoxia tolerance contributing to ecological niche specialization between species (11)]. The preferential expression of cTnC in trout may be indicative of the role of cTnC relative to ssTnC. Temperature has been suggested to be a potential reason for increased Ca^2+^ affinity in fish cTnC relative to mammals. The increased Ca^2+^ affinity of trout cTnC allows for function at colder temperatures (4, 20). Therefore, regardless of the chamber, cTnC may be needed for adequate contraction of the heart at lower temperatures (e.g., 5°C), and thus species that live at colder temperatures may express higher innate levels of this isoform.

#### Temperature-specific paralog profiles.

To explore further the variation in isoform profiles, both species of fish were exposed to an environmental perturbation to determine if the isoforms were differentially sensitive. Because cTnC is known to be temperature sensitive, as well as the fact that the temperature conditions of the two different study species vary by temperature, fish were subjected to a temperature acclimation regime. Zebrafish were cold acclimated, and trout were warm acclimated by 10°C. This 10°C difference in temperature was adequate to induce a significant shift in paralog expression in the zebrafish and trout (Fig. 5). The overall trend of cTnC being predominant in ventricles and at colder temperatures appears in both species. In zebrafish, both the atrium and ventricle express more cTnC than ssTnC at 18°C than at 28°C. In trout, both chambers still express more cTnC than ssTnC at 15°C, but ssTnC expression is much greater at 15°C than at 5°C. However, there appears to be phylogenetically different responses. In zebrafish levels of cTnC decrease in both chambers with decreased temperature; in trout, levels of cTnC remain relatively constant and only ssTnC fluctuate.

Temperature adaptive shifting in gene expression is the basis for physiological acclimation (42). Interestingly, both the paralog profile and the responses to changing temperature do not match with the two species of fish examined here. This suggests a phylogenic-specific pattern in usage of TnC paralogs. While ssTnC levels increase with temperature in the atrium of both species, the scope relative to cTnC transcript levels is not the same. In fact, the transcript levels of cTnC vary in zebrafish, whereas they remain relatively constant in trout. The evolutionary history of environmental temperature may play a role in how each species remodels the cardiac chambers, or regulates the transcriptional levels of contractile proteins.

### Summary

The subfunctionalization of TnC isoforms demonstrated in this study lends insight into the consequences of variation to the structure-function of cTn and variation in cardiac muscle contraction. Due to this novel fish-specific paralog, the temperature-dependent phenotype of the fish heart may be more complex than previously thought, especially when coupled to the chamber-specific differences in expression and responsiveness to temperature seen in zebrafish. Variation in isoforms of sarcomeric proteins influencing the chamber-specific contractile properties may be important in achieving physiological versatility and hence the evolution of specializations in the chambers of the fish heart.

## GRANTS

This work was supported by grants from the Natural Sciences and Engineering Research Council of Canada and the Heart and Stroke Foundation of Canada to G. F. Tibbits. G. F. Tibbits is the recipient of a Tier I Canada Research Chair.

## DISCLOSURES

No conflicts of interest, financial or otherwise, are declared by the author(s).

## AUTHOR CONTRIBUTIONS

Author contributions: C.E.G. and G.F.T. conception and design of research; C.E.G. performed experiments; C.E.G. analyzed data; C.E.G., W.S.D., and G.F.T. interpreted results of experiments; C.E.G. prepared figures; C.E.G. drafted manuscript; C.E.G., W.S.D., and G.F.T. edited and revised manuscript; W.S.D. and G.F.T. approved final version of manuscript.
